# Nanoparticulate Immunoactive Complex for Local Chemoimmunotherapy: From Murine Models to Pilot Canine Study

**DOI:** 10.1158/2767-9764.CRC-26-0110

**Published:** 2026-06-22

**Authors:** Yanying He, Jiaqi Zhao, Jung Suk Kim, Meaghan M. Broman, Simone de Brot, Fanfei Meng, Deepika Dhawan, Sagar M. Utturkar, Marejka H. Shaevitz, Lindsey M. Fourez, Deborah W. Knapp, Yoon Yeo

**Affiliations:** 1 Department of Industrial and Molecular Pharmaceutics, Purdue University, West Lafayette, Indiana.; 2 Department of Comparative Pathobiology, College of Veterinary Medicine, Purdue University, West Lafayette, Indiana.; 3 Institute of Animal Pathology, COMPATH, University of Bern, Bern, Switzerland.; 4 Department of Pharmacological and Pharmaceutical Sciences, College of Pharmacy, University of Houston, Houston, Texas.; 5 Department of Veterinary Clinical Sciences, College of Veterinary Medicine, Purdue University, West Lafayette, Indiana.; 6 Purdue University Institute for Cancer Research, West Lafayette, Indiana.; 7 Weldon School of Biomedical Engineering, Purdue University, West Lafayette, Indiana.

## Abstract

Chemoimmunotherapy leverages the immune system to control tumor growth and develop durable antitumor immunity. With this goal, we previously developed a nanoparticulate immunoactive complex (IMAX), composed of polyethyleneimine–lithocholic acid conjugate, paclitaxel, and a cyclic dinucleotide, which showed robust antitumor activity in multiple mouse models following intratumoral administration. In this study, we evaluated the biodistribution of locally delivered IMAX and determined a safe and effective dose in mice to inform pilot safety and efficacy evaluations in dogs. In mice, IMAX remained localized at the injection site with minimal systemic exposure, induced rapid immune cell infiltration, evidenced by gross skin reactions and histologic changes, and achieved complete tumor regression even at one-fifth of the initial dose. Laboratory dogs tolerated IMAX well, with only transient, manageable adverse events. In a pilot study in companion dogs with naturally occurring cancer, IMAX administered at conservative doses induced histologic changes indicating immune cell infiltration and necrosis, accompanied by transcriptomic signatures of early innate immune pathway activation, resulting in measurable antitumor effects in the dogs with mammary carcinoma. These findings support the feasibility and safety of local IMAX therapy in tumor-bearing dogs and warrant further investigation with optimized dosing and combination strategies.

**Significance:**

Locally administered nanoparticulate IMAX induces complete tumor regression in mice and triggers rapid immune cell infiltration, while producing measurable antitumor responses in companion dogs with mammary carcinoma, supporting the translational potential.

## Introduction

Cancer immunotherapy harnesses the immune system to control tumor growth, induce regression of established tumors, and develop long-lasting antitumor immune memory to prevent recurrence. Toward this goal, numerous preclinical and clinical studies have investigated combinations of chemotherapy and immunotherapy—an approach known as chemoimmunotherapy. The rationale behind this strategy is that chemotherapy can induce immunogenic cell death (ICD) in tumor cells, generating tumor antigens and intrinsic immunoadjuvants called damage-associated molecular patterns (DAMP; ref. 1), while immunostimulatory agents serve as extrinsic immunoadjuvants. The therapy-induced antigens and immunoadjuvants recruit and activate innate immune cells, recognize tumor antigens, and trigger the innate immune response to tumors, translating to tumor-specific adaptive immune responses.

Building on this rationale, we previously developed a nanoparticulate immunoactive complex (IMAX) for local treatment of accessible tumors, such as melanoma and, head and neck cancer, or microscopic residual tumors following surgical resection. IMAX consists of a polyethylenimeine–lithocholic acid conjugate (2E′), paclitaxel (PTX), and cyclic dinucleotide (CDN; ref. 2). 2E′ self-assembles into nanoparticles, in which PTX is encapsulated in the hydrophobic lithocholic acid core, and CDN binds to the cationic polyethyleneimine surface. These components have multiple functions in the tumor microenvironment, which collectively stimulate the innate immune response to tumors. 2E′ activates Toll-like receptor 5 (TLR5) and inflammasome to activate antigen-presenting cells (APC) and selectively kills tumor cells (2). PTX induces cell-cycle arrest in proliferating cells, causing endoplasmic reticulum stress (3). In our previous study, we validated PTX as an ICD inducer by showing that PTX-treated tumor cells release classic DAMPs during cell death and that the PTX-killed tumor cells can effectively function as a tumor vaccine (4). CDN, a stimulator of interferon genes protein (STING) agonist, induces the production of proinflammatory cytokines, including type 1 interferons (IFN) by APCs and tumor cells (5–7). 2E′ has proven to be an effective carrier of PTX and CDN, which are otherwise difficult to codeliver due to their distinct chemical properties. Through these activities, IMAX showed impressive antitumor activity in different mouse models of ectopic tumors, leading to tumor-free survival in 80% to 100% of treated mice after a single intratumoral administration (2, 8). Additionally, the mice surviving with complete tumor regression rejected a rechallenge with the same tumor cells (but not unrelated tumor cells), indicating the induction of specific antitumor immune memory (2, 8). These results indicate that IMAX is an effective strategy to induce antitumor immune responses.

Encouraged by these compelling preclinical results, in this study we have advanced to evaluate the safety and preliminary efficacy of IMAX in dogs as the first step toward clinical translation to humans. Specific types of naturally occurring cancer in companion dogs are attractive for drug development research because of their similarities to human cancer in histopathologic, cellular, molecular and immunologic features, as well as in inherent and acquired drug resistance paralleling that in humans (9, 10). Moreover, because companion dogs share environmental and other exposures with humans, they provide a relevant model for disease pathogenesis. We first examined the biodistribution of IMAX, represented by PTX, and tissue responses to locally administered IMAX in mice, established a minimum effective dose to guide dog studies, and evaluated the safety of IMAX in three laboratory dogs. We then investigated therapeutic activity and longitudinal pathologic and molecular changes with IMAX in a pilot study with four companion dogs—two with mammary carcinoma and two with soft tissue sarcoma, both of which display similarities in presentation, clinical history, molecular profiles, response to therapy, and patterns of resistance to their human counterparts (11–18). Our results demonstrate that IMAX can be safely administered to mice and dogs, inducing measurable antitumor effects, accompanied by innate immune activation.

## Materials and Methods

### Materials

Polyethyleneimine (MW 2500) was purchased from Kyfora Bio, and CDN [2′3′-c-di-AM(PS)2 (Rp,Rp)] was purchased from Invitrogen. Paclitaxel was purchased from Biotang. Docetaxel was purchased from Thermo Fisher Scientific. LEGENDplex Mouse Anti-Virus Response Panel was purchased from BioLegend.

### IMAX preparation and characterization

IMAX (2E′/PTX/CDN, 1:0.2:0.02 wt. ratio) was prepared by the membrane hydration method as reported previously (2, 8). First, 2E′ was synthesized as previously reported (2). 2E′ and PTX were first dissolved in a mixture of ethanol and chloroform (1.5:3.5 v/v) in a round-bottom flask. The solvent was evaporated using a rotary evaporator to form a thin film. Deionized (DI) water was added to the flask to hydrate the film, which was dispersed by a probe sonicator (20% amplitude, 1 second on/1 second off, 1 minute) to form a 2E′/PTX binary complex. CDN solution was added to the 2E′/PTX suspension and incubated for 20 minutes to make a 2E′/PTX/CDN triple complex (IMAX). Z-average and zeta-potential of IMAX were measured using the Malvern Zetasizer Nano ZS90. IMAX was dispersed in 5% dextrose solution (D5W) at a concentration equivalent to 0.05 to 10 mg/mL 2E′ (for mice) and 2 mg/mL 2E′ (for dogs), unless specified otherwise. IMAX was used freshly or lyophilized using a Labconco benchtop Freeze Dyer (Freezone 4.5L −84°C) for 2 days. The lyophilized IMAX was stored in −20°C and was reconstituted in DI water.

### Animal care

All animal procedures were approved by the Institutional Animal Care and Use Committee in conformity with the NIH guidelines for animal studies. BALB/c mice (RRID: MGI:2683685) were purchased from Envigo and acclimatized for 1 week before the procedure. Laboratory beagle dogs were obtained from Marshall Bioresources, acclimatized for 2 weeks, and housed in individual runs. Companion dogs with naturally occurring external tumors, who had failed prior therapies or were not eligible for other therapies, were recruited from cases presented to the Purdue Veterinary Hospital for cancer care. The companion dogs were enrolled with the informed consent of their owners, continued to live in private homes as pets, and presented to the hospital for treatment and evaluation.

### Biodistribution of PTX in mice by local IMAX injection

CT26 tumor-bearing mouse models were established by inoculating 5 × 10^5^ CT26 mouse colon carcinoma cells (AATC, RRID: CVCL_7254) subcutaneously in the upper flank of the right hind limb of 7- to 8-week-old female BALB/c mice. CT26 cells were used as received from the vendor (AATC) without additional testing. Tumors were grown to a relatively small volume of 30 to 50 mm^3^ to model a postsurgical residual disease. Once tumors reached the target volume, the mice were treated with an intratumoral injection of D5W or IMAX (consisting of 2E′ 1 mg, PTX 0.2 mg, and CDN 0.02 mg). Non–tumor-bearing healthy BALB/c mice received subcutaneous injections of D5W or IMAX (consisting of 2E′ 1 mg, PTX 0.2 mg, and CDN 0.02 mg). Mice were sacrificed at 6, 24, and 48 hours after injection (four mice per time point). Skin at the injection site was harvested and weighed, and whole blood was collected via cardiac puncture and stored in an EDTA-coated tube.

Skin tissues were homogenized in cold phosphate-buffered saline (PBS) at 1 g tissue per 4 mL PBS using an Omni Tissue Master 125 homogenizer. Tissue homogenates (0.2 mL) were spiked with 600 ng of docetaxel (DTX, internal standard) and extracted with 0.8 mL of methyl tert-butyl ether (MTBE) for 1.5 hours. Calibration standards were prepared with tissue homogenates with known quantities of PTX (128, 64, 32, 16, 8, and 4 μg/mL) and 3 μg/mL of DTX, processed in the same way. After centrifugation at 10,000 × *g* for 10 minutes, 0.6 mL of the organic phase was collected, evaporated, and reconstituted in 60 μL of acetonitrile (ACN) for liquid chromatography–tandem mass spectrometry (LC-MS/MS) analysis. Whole blood was centrifuged at 1,000 × *g* for 10 minutes to separate plasma. PTX was extracted from 0.2 mL plasma samples in the same way as tissue homogenates. Plasma calibration standards were prepared by plasma containing PTX at concentrations of 500, 250, 125, 62.5, 31.3, 15.6, 7.8, and 3.9 ng/mL, along with 3 μg/mL of DTX.

PTX was quantified using a 6460 QQQ LC/MS/MS mass spectrometer (Agilent Technologies). Ten microliters of sample or standard was injected into an Xbridge C18 column (2.1 mm × 100 mm, particle size 3.5 μm, Waters Corporation) maintained at 35°C. The mobile phase consisted of 0.1% formic acid (FA) in water (A) and 0.1% FA in methanol (B), run at a flow rate of 0.3 mL/minute over a 15-minute gradient: A/B (50/50) at 1 minute, ramped to A/B (0/100) by 11 minutes, and returned to A/B (50/50) by 15 minutes. The parent ions, (PTX + H)^+^ and (DTX + H)^+^, were monitored at the m/z ratios of 286.2 and 327, respectively. PTX concentrations were determined based on tissue-specific calibration curves plotting PTX/DTX peak area ratios against standard concentrations.

### Measurement of plasma cytokine levels in mice following local IMAX injection

Blood samples were collected from the submandibular vein of both healthy and CT26 tumor-bearing BALB/c mice 1 hour after treatment with IMAX (consisting of 2E′ 1 mg, PTX 0.2 mg, and CDN 0.02 mg). Collected blood was centrifuged at 1,000 × *g* for 10 minutes to separate plasma, which was stored at −80°C until analysis. Cytokine levels in the plasma were determined by the LEGENDplex Mouse Anti-Virus Response Panel (13-plex, BioLegend; cat. #740621) following the manufacturer’s instructions. Samples were analyzed by the Attune flow cytometer (Thermo Fisher Scientific), and cytokine concentrations were calculated by the BioLegend LEGENDplex Data Analysis program, based on standard curves generated from the internal standards.

### Skin histology in mice following local IMAX injection

Part of the skin samples collected at the sacrifice for the biodistribution study (at 6, 24, and 48 hours after injection) were processed for histologic examination. Additional groups of mice (healthy BALB/c mice and CT26 tumor-bearing mice) were treated in the same way (IMAX consisting of 2E′ 1 mg, PTX 0.2 mg, and CDN 0.02 mg injected subcutaneously or intratumorally) and sacrificed at 9 days after treatment for collecting skin at the injection site. The collected skin samples were fixed in 4% paraformaldehyde in PBS, paraffin-embedded, sectioned at a thickness of 7 μm, and subjected to hematoxylin and eosin (H&E) and Gram staining. Whole-slide digital images were acquired using a Leica Versa8 scanner.

### Dose-dependent skin response of IMAX in healthy and CT26 tumor-bearing BALB/c mice

CT26 tumor cells (5 × 10^5^) were inoculated subcutaneously in the upper flank of the right hind limb of BALB/c mice. When the tumor size reached 30 to 50 mm^3^, each mouse received an intratumoral injection of IMAX at a dose equivalent to 1, 0.5, or 0.2 mg of 2E′ with corresponding amounts of PTX and CDN (2E′/PTX/CDN, at 1:0.2:0.02 wt. ratio; *n* = 5 per dose). Healthy BALB/c mice were administered the same doses (*n* = 3 per dose) via subcutaneous injection. Scab formation at the injection site was monitored every other day, and the length (*l*) and the width (*w*) of the scab were measured using a digital caliper. The scab area was calculated according to the ellipse area formula: (l×w×π)/4.

### Determination of the minimum effective dose of IMAX in BALB/c mice with CT26 tumors

CT26 tumor models were established as described above. When the tumor grew to 30 to 50 mm^3^, mice were randomized into six groups and treated with an intratumoral injection of IMAX equivalent to 1, 0.5, 0.2, 0.05, or 0.005 mg 2E′ with corresponding amounts of PTX and CDN (2E′/PTX/CDN, at 1:0.2:0.02 wt. ratio) or PBS as a control (*n* = 5 per dose). Tumor growth was monitored over 80 days, with the length (*L*) and the width (*W*) of the tumor measured using a digital caliper. Tumor volumes were calculated according to the modified ellipsoid formula: $\left(W^{2}\times L\right)/2$.

### Safety and dose evaluation study in laboratory dogs

The laboratory dog study was performed in the Pre-Clinical Research Laboratory in the Center for Clinical Translational Research in the Purdue University College of Veterinary Medicine. IMAX was tested in three beagle dogs approximately 1 year of age, including one female (dog 1W) and two male dogs (dogs 2P and 3C). As tumor masses were absent in the normal dogs, IMAX was injected subcutaneously on the side of the body. Table 1 lists the administered doses. Two dogs (dogs 1W and 2P) received 1 mL of IMAX subcutaneously on the right and left sides of the body. Each dog received an IMAX dose containing 2E′ 2 mg, PTX 0.4 mg, and CDN 0.04 mg on the right side and an IMAX dose containing 2E′ 1 mg, PTX 0.2 mg, and CDN 0.02 mg on the left side. After evaluating the effect of the first injection, we administered a second IMAX injection at 13 days (dog 1W) and 21 days (dog 2P) from the first dose. The second injection contained 2E′ 2 mg, PTX 0.4 mg, and CDN 0.04 mg and was administered subcutaneously on one side. The third dog (dog 3C) received a single subcutaneous injection of IMAX containing 2E′ 2 mg, PTX 0.4 mg, and CDN 0.04 mg on the right side. Dexamethasone (0.1 mg/kg s.c.) and diphenhydramine (2 mg/kg s.c.) were administered to 1W and 2P when the body temperature rose above 103°F and the dogs showed signs concerning the development of an anaphylactic-like reaction. With subsequent treatments, however, the onset, level, and resolution of the increased body temperature were similar even when dexamethasone and diphenhydramine were omitted.

**Table 1.** IMAX regimen for laboratory safety study in beagle dogs.

Subject	Subject detail	First dose - right	First dose - left	Second dose
1W	F, beagle, 7.8 kg	2E′: 2 mg PTX: 0.4 mg CDN: 0.04 mg	2E′: 1 mg PTX: 0.2 mg CDN: 0.02 mg	2E′: 2 mg PTX: 0.4 mg CDN: 0.04 mg 13 days after the first dose
2P	M, beagle, 11.4 kg	2E′: 2 mg PTX: 0.4 mg CDN: 0.04 mg	2E′: 1 mg PTX: 0.2 mg CDN: 0.02 mg	2E′: 2 mg PTX: 0.4 mg CDN: 0.04 mg 21 days after the first dose
3C	M, beagle, 11.2 kg	2E′: 2 mg PTX: 0.4 mg CDN: 0.04 mg	Not applicable	Not applicable

Adverse events were assessed by (i) inspection of the injection site and monitoring temperature, pulse, and respiration daily for 7 days and then once weekly after each injection; (ii) complete physical exam before injection and then daily for 5 days; and (iii) complete blood count and serum biochemical profile before injection and at 2 weeks after injection. The dogs were monitored more frequently when inflammation or adverse events were observed. Adverse events were recorded and classified according to Veterinary Cooperative Oncology Group (VCOG) criteria (19). Following participation in the study, the dogs were adopted into private homes to continue life as companion animals.

### Pilot study of IMAX activity in companion dogs

A pilot study of IMAX in companion dogs with naturally occurring tumors was performed to determine the antitumor effects and changes in tumor tissue longitudinally over time. The study was performed at the Purdue University Veterinary Hospital by veterinarians and staff of the Werling Comparative Oncology Research Center. Inclusion criteria were (i) the presence of naturally occurring histopathologically confirmed cancer (excluding mast cell tumors) on the outside of the dog’s body accessible for injection and biopsy, (ii) no major organ dysfunction, (iii) expected survival of at least 6 weeks, and (iv) those that had failed or were not eligible for other therapies or for which the dog owners had declined other therapies.

Four companion dogs were enrolled with the informed consent of their owners. As untreated cancer can progress rapidly, it is unethical to have an untreated control group of companion dogs. Instead, we used pretreatment measurements as each dog’s baseline to evaluate changes in this pilot study. The primary tumor was measured in three dimensions (cranial–caudal, medial–lateral, and dorsal–ventral) using calipers before treatment and weekly following treatment. The longest dimension was used to determine the response according to RECIST criteria (19, 20). Tumor response was evaluated according to the iRECIST criteria (20), applied to the VCOG RESIST1.0 standard (21). Briefly, the sum of the longest dimensions of tumor lesions for each dog (one lesion in two dogs and two lesions in two dogs) was recorded over time. A 20% increase in size from baseline or development of new lesions was considered progressive disease (PD, per RECIST), 30% decrease in size without the development of new lesions was considered partial response (PR, per RECIST), a change of less than 20% increase or 30% decrease without the development of new lesions was considered stable disease (SD, per RECIST), and complete resolution of all measurable lesions without the development of new lesions was considered a complete response (CR, per RECIST). iRECIST had the same criteria as RECIST, except for the addition of the unconfirmed PD (iUPD)—potentially due to pseudoprogression—and confirmed PD (iCPD) categories (20). Tumor volumes were also recorded according to the modified ellipsoid formula as $\left(W^{2}\times L\right)/2$, with *W* defined as the shortest measured dimension and *L* defined as the longest measured dimension. Cancer stage was assessed with thoracic and abdominal radiography, abdominal ultrasonography, and physical examination. In the dog weighing >40 kg, a whole-body CT scan was performed.

Each dog received two to three IMAX treatments, with the second dose given 3 to 4 weeks after the initial dose. A third dose was allowed if there was evidence of tumor control or shrinkage in response to the treatment (Table 2). IMAX was administered intratumorally at a dose found to be safe in laboratory dogs. The dose was based on body weight, irrespective of tumor size (2E′ 2 mg, PTX 0.4 mg, and CDN 0.04 mg per 7.8 kg = 2E′ 0.25 mg/kg, PTX 0.05 mg/kg, and CDN 0.005 mg/kg). Body weight–based dosing was performed to ensure the dose would be tolerable should the drug unexpectedly enter the systemic circulation through the tumor vasculature. IMAX was dispersed in D5W at a concentration 2E′ 2 mg/mL, PTX 0.4 mg/mL, and CDN 0.04 mg/mL and injected in one or two locations within the tumor mass. The dogs were sedated or anesthetized for the injection.

**Table 2.** Subject and tumor characteristics and IMAX treatments in companion dogs in the pilot study.

Subject - dog	Subject detail	Tumor; volume	First dose	Second dose	Third dose
RV	FS, mixed breed, 22.3 kg, 9.7 years old	Soft tissue sarcoma (right lateral hock); 7.6 cm^3^	2.2 mL 2E′: 4.4 mg PTX: 0.88 mg CDN: 0.088 mg	2.7 mL 2E′: 5.4 mg PTX: 1.08 mg CDN: 0.108 mg 21 days after the first dose	2.8 mL 2E′: 5.6 mg PTX: 1.12 mg CDN: 0.112 mg 57 days after the first dose
SP	FS, Labrador Retriever, 30 kg, 7.2 years old	Soft tissue sarcoma (left palmar carpus); 25.7 cm^3^	4.2 mL 2E′: 8.4 mg PTX: 1.68 mg CDN: 0.168 mg	4 mL 2E′: 8 mg PTX: 1.68 mg CDN: 0.168 mg 23 days after the first dose	4 mL 2E′: 8 mg PTX: 1.68 mg CDN: 0.168 mg 86 days after the first dose
CL	FI, Shih Tzu, 5 kg, 13.7 years old	Mammary carcinoma (left mammary chain); 0.3 and 1 cm^3^	0.6 mL (0.3 mL in each tumor) 2E′: 1.2 mg PTX: 0.24 mg CDN: 0.024 mg	0.7 mL (0.25 mL for smaller tumor; 0.45 mL for larger tumor) 2E′: 1.4 mg PTX: 0.28 mg CDN: 0.028 mg 20 days after the first dose	Not applicable
BM	FS, Great Pyrenees, 43.2 kg, 9.7 years old	Mammary carcinoma (both caudal mammary glands); 36.3 and 3.1 cm^3^	5.4 mL (to the larger tumor only) 2E′: 10.8 mg PTX: 2.16 mg CDN: 0.216 mg	5.5 mL (to the larger tumor only) 2E′: 11 mg PTX: 2.2 mg CDN: 0.22 mg 28 days after the first dose	Not applicable

Abbreviations: FI, female intact; FS, female spayed.

Body temperature was checked at 2 and 4 hours after injection and then at 4- to 6-hour intervals. The dogs were monitored in-hospital overnight following injections and then allowed to go home the next day if no grade 3 or higher adverse events occurred. Dexamethasone (0.1 mg/kg s.c. or i.v.) and diphenhydramine (2 mg/kg s.c. or i.m.) were available for dogs showing signs of anaphylactic-type reactions, but none of the dogs required these medications. Physical examination was performed weekly during IMAX treatment. Complete blood counts and serum biochemical profiles were assessed before treatment and at monthly intervals during treatment. Adverse events were classified according to VCOG criteria. At the conclusion of the study, the primary tumors were removed surgically with complete margins in all four dogs.

Tumor biopsies were obtained before and after treatment with the intent to obtain posttreatment biopsies at the time of tumor shrinkage. Following the initial dose, the change in tumor size was monitored. This time course was used to select the timing of the biopsy following the second treatment. Thus, the timing of posttreatment biopsies varied from dog to dog. Following 2 to 3 doses of IMAX, the tumors were surgically removed as appropriate to prevent local progression and potential metastasis. Tissues were also collected at the time of surgical removal of the tumor. Histology, immunohistochemistry (IHC), and RNA sequencing analysis (RNA-seq) were performed to evaluate immune cell infiltration in the tumor.

### Companion dog histology and IHC

Biopsy specimens were collected from the companion dogs at the Purdue University Veterinary Hospital, fixed in 10% neutral-buffered formalin, and submitted to the Willie M. Reed Animal Disease Diagnostic Laboratory for paraffin embedding, sectioning, H&E staining, and IHC. IHC antibodies and abbreviated protocols are listed in Supplementary Table S1. Whole-slide digital images were acquired using a Leica Versa8 scanner.

### RNA-seq analysis of dog biopsies

Tumor samples from companion dogs were collected in TRIzol and stored at −80°C. RNA was isolated using the AllPrep DNA/RNA Kit (Qiagen) following the manufacturer’s instructions. The isolated RNA was submitted for strand-specific RNA-seq (Azenta Life Sciences) to yield 30 to 40 million reads per sample. Raw data were aligned to CanFam4.0 using Strand (Strand NGS) and normalized using TMM and edgeR (RRID: SCR_012802; ref. 22). Unsupervised hierarchical clustering was performed on normalized counts to generate heatmaps using manually curated gene lists of pathways of interest to the study (23). Data were scaled across samples to evaluate posttreatment trends in genes/pathways. The posttreatment samples were compared with the pretreatment sample for each dog. Normalized RNA-seq data were also used to run single-sample gene set enrichment analysis (ssGSEA; Broad Institute, MIT; RRID: SCR_026610). The gene sets used for enrichment analyses were manually curated based on interest of the study (23–25). Positive and negative ssGSEA scores were determined across samples for each pathway. These scores are relative and used to compare the activity of the pathway across samples and evaluate trends within samples after IMAX treatment. The scores were represented as separate heatmaps for each treated dog.

### Statistical analysis

Statistical analysis was performed using the GraphPad Prism 9 (RRID: SCR_002798). Data were analyzed by a two-way ANOVA test to determine the statistical difference among groups, followed by Sidak multiple comparisons test. A *P* value of <0.05 was considered statistically significant. Quantitative data were presented as the mean ± standard deviation (SD).

## Results

### Locally injected IMAX was retained at the injection site with minimal systemic absorption in mice

IMAX showed a z-average of 287.3 ± 10.4 nm with polydispersity index of 0.284 ± 0.05 and surface charge of 21.2 ± 1.2 mV, measured by dynamic light scattering. As IMAX was prepared with a purified polymer and the organic solvent was removed by evaporation, the formulation was used as prepared without additional purification. Therefore, the drug loading corresponds to the nominal input ratio (2E′/PTX/CDN = 1:0.2:0.02 wt. Ratio). IMAX could be lyophilized, and the lyophilized IMAX maintained the size, zeta-potential, morphology, and bioactivity after storage at −20°C for at least 4 months (8).

**Figure 1.**
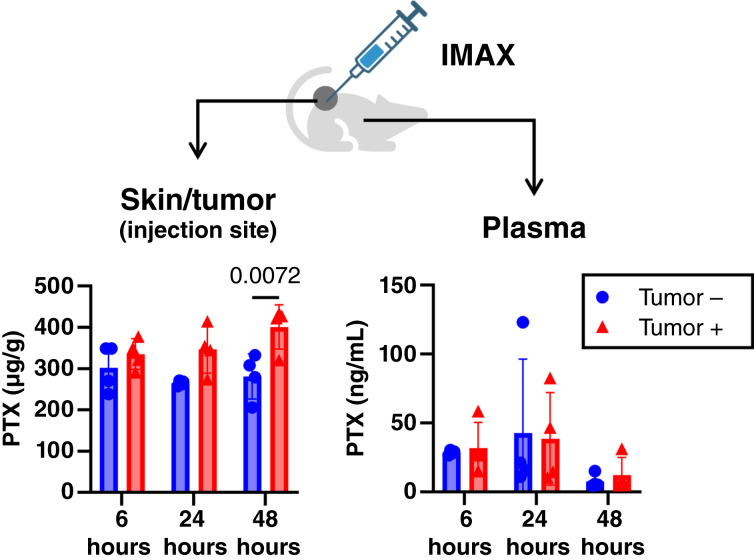
Locally injected IMAX is retained at the injection site with minimal systemic absorption. PTX levels (μg/g) in skin (or tumor) and (ng/mL) in plasma at 6, 24, and 48 hours after subcutaneous or intratumoral injection of IMAX (containing 1 mg 2E′) in healthy and CT26 tumor-bearing BALB/c mice (7–8 weeks). *n* = 4 mice per time point. Data are the mean ± SD. *P* value was calculated by Sidak multiple comparisons test, following two-way ANOVA.

To evaluate the retention and systemic absorption of locally injected IMAX, we examined the level of PTX—a component of IMAX—at the injection site and in blood at 6, 24, and 48 hours after injection (intratumorally for tumor-bearing mouse and subcutaneously for healthy mouse; Fig. 1; Supplementary Fig. S1). Healthy and tumor-bearing mice showed plasma PTX levels of 43 and 39 ng/mL on the average (<0.06 % injected dose/g, %ID/g) over 24 hours, with the levels declining to 8 and 12 ng/mL (0.004 and 0.006 %ID/g) by 48 hours, respectively. In contrast, PTX was present at 281 and 401 μg/g on the average (140 and 200 %ID/g) at the injection site in healthy and tumor-bearing mice over 48 hours, respectively. This result indicates that locally injected IMAX was retained at the injection site with minimal systemic absorption, also consistent with the retention of another component (CDN) of IMAX in tumor-bearing mice (8). Interestingly, PTX levels at the injection site at 48 hours were higher in CT26 tumor-bearing mice than healthy mice, suggesting greater IMAX retention in tumors than in normal skin, attributable to impaired lymphatic drainage in rapidly growing tumors (26, 27).

### Locally injected IMAX did not alter systemic cytokine profiles, reflecting minimal systemic absorption in mice

**Figure 2.**
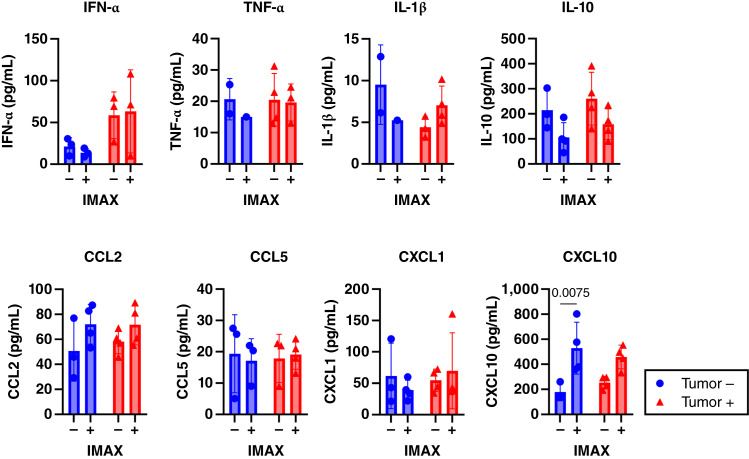
Locally injected IMAX does not alter systemic cytokine profiles reflecting minimal systemic absorption. Plasma levels of IFN-α, TNF-α, IL-1β, IL-10, CCL2, CCL5, CXCL1, and CXCL10 at 1 hour after subcutaneous or intratumoral injection of IMAX (containing 1 mg 2E′) in healthy and CT26 tumor-bearing BALB/c mice (7–8 weeks). *n* = 4 mice per group. Data are the mean ± SD. *P* value was calculated by Sidak multiple comparisons test, following two-way ANOVA.

To test the immediate systemic effect of IMAX, we measured circulating cytokine levels in the treated mice at 1 hour after IMAX injection (Fig. 2). We selected this time point based on the well-established cytokine kinetics, where proinflammatory cytokines rapidly reach peak levels within 1 to 3 hours following stimulation and subside thereafter (28). Two components of IMAX—2E′ and CDN—are agonists of TLR5 and STING pathways, respectively, and known to increase the production of CXCL9, CXCL10 (29), interleukins (IL), types I and II IFN, and tumor necrosis factor (TNF)-α (30, 31). Nevertheless, the locally injected IMAX did not increase the levels of cytokine/chemokines in the blood. Except for CXCL10, none of the examined proinflammatory chemokines or cytokines (IFN-α, TNF-α, IL-1β, CCL2, CCL5, and CXCL1) were elevated at 1 hour following local injection of IMAX in both healthy and tumor-bearing mice. This result is consistent with the biodistribution study, which indicates minimal drug absorption into the system following local IMAX injection.

### Locally retained IMAX induced time-dependent changes in the (immune) cell population at the injection site in mice

**Figure 3.**
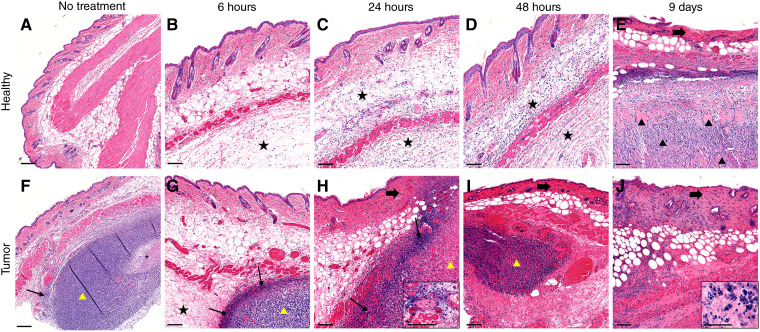
Skin histology of healthy or CT26 tumor-bearing BALB/c mice before and after IMAX treatment. **A,** Healthy mouse skin before treatment. **B,** At 6 hours after treatment, healthy mice develop subcutaneous edema and neutrophilic infiltration (★). **C,** At 24 hours after treatment and (**D**) 48 hours after treatment, subcutaneous edema and inflammation (★) remain. **E,** At 9 days after treatment, the epidermis and dermis are necrotic (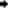). Necrosis extends to the subcutis with areas of fibroblast proliferation and skeletal muscle necrosis and regeneration (▲). **F,** Tumor-bearing mouse before treatment. Note central necrosis (*) in tumor (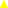) and leukocytes infiltrating the peritumoral connective tissue (→). **G,** At 6 hours after treatment, tumor-bearing mice develop subcutaneous and peritumoral edema and neutrophilic infiltration (★) similar to healthy mice. Note leukocyte infiltration (→) at the periphery of the tumor (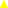). **H,** At 24 hours after treatment, the tumor (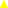) is extensively necrotic with marked peritumoral leukocytic infiltration (→). The overlying dermis is hypereosinophilic and compacted (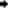). Note extracellular blue-stained granular materials in necrotic areas (inset). **I,** At 48 hours after treatment, the tumor (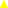) is necrotic and the overlying dermis is hypereosinophilic and necrotic (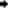). **J,** At 9 days after treatment, the epidermis and dermis are necrotic (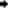). Intracellular and extracellular blue materials are also present (inset). Scale bars, 100 μm.

We examined temporal changes of tissue at the injection site in mice to gain mechanistic insights into IMAX’s antitumor effects (Fig. 3; Supplementary Fig. S2). Following subcutaneous or IT IMAX injection (IMAX consisting of 2E′ 1 mg, PTX 0.2 mg, and CDN 0.02 mg), a local skin response appeared grossly from 1 day after injection, developing a scab over the next few days. Mild swelling was observed at the injection site as early as 6 hours after injection and persisted over 24 hours. By 24 hours after the injection, mild erythema appeared in most mice, more prominently in the tumor-bearing mice. The swelling subsided by 48 hours, but the area of erythema slightly increased, appearing darker in the tumor-bearing mice, with small scabs forming at the injection site in most animals. By 9 days after injection, the injection site was mostly covered with dry scabs.

Histologic changes in healthy mice at 6 hours after IMAX treatment, compared with untreated control (Fig. 3A), included local hyperemia, moderate to marked edema, and mild to moderate neutrophilic infiltration within the subcutis (Fig. 3B). Hyperemia, edema, and inflammation progressed in severity at the 24- and 48-hour posttreatment time points, with numerous lymphocytes infiltrating the subcutis (Fig. 3C and D). At 9 days after treatment, full-thickness epidermal and dermal necrosis with necrotic neutrophils was present, with inflammation, fibrin, and necrosis extending into the surrounding connective tissues and underlying skeletal muscle (Fig. 3E). Some sections included areas of fibroblast proliferation and skeletal muscle necrosis and regeneration (Fig. 3E) and scattered, blue-stained extracellular granular materials. In some areas, the epidermis was ulcerated and replaced by a serocellular crust composed of necrotic neutrophils, serous fluid, and erythrocytes, corresponding to the scabbing observed grossly.

Tumors receiving IMAX showed comparable changes. Untreated tumors consisted of elongate to spindloid cells within a scant fibrovascular stroma with central necrosis (Fig. 3F). Neutrophils and fewer lymphocytes were present at the tumor periphery, and few neutrophils infiltrated the tumor mass (Fig. 3F). At 6 hours after IMAX treatment, subcutaneous edema and neutrophilic infiltration were observed similar to those in the healthy mice (Fig. 3G). Neutrophils, macrophages, and few lymphocytes infiltrated the peritumoral connective tissues (Fig. 3G). By 24 hours, in addition to increased subcutaneous edema and inflammatory cell infiltration, tumor tissue was markedly necrotic with scattered, blue-stained extracellular granular materials within necrotic areas (Fig. 3H). At 48 hours, increased inflammatory cells, including numerous neutrophils and macrophages, infiltrated the subcutis, and many tumor cells were shrunken with small dark pyknotic nuclei (apoptosis). The blue-stained granular materials were both extracellular and intrahistiocytic (Fig. 3I). Additionally, there was marked necrosis of the overlying epidermis and dermis (Fig. 3I). At 9 days after treatment, tumor tissue was not identified in most sections. They were similar to the healthy mice at 9 days after treatment, with full-thickness epidermal/dermal necrosis and crusting, along with inflammation, as well as necrosis of the subcutaneous connective tissues and muscle (Fig. 3J). The blue-stained granular material was mostly intrahistiocytic and abundant in areas of necrosis (Fig. 3J).

Overall, IMAX treatment induced quick infiltration of innate immune cells, resulting in tumor necrosis and regression, as well as locally extensive necrosis of the surrounding skin, connective tissues, and skeletal muscle in mice. The composition of the blue granular material is uncertain. Gram staining did not reveal Gram-positive bacteria (Supplementary Fig. S3), ruling out bacterial contamination. As this material was observed after treatment, mainly in areas of necrosis, in both healthy and tumor-bearing mice, we speculate that it represents nuclear debris from necrotic cells.

### The extent of local skin response to IMAX was dose-dependent in mice

**Figure 4.**
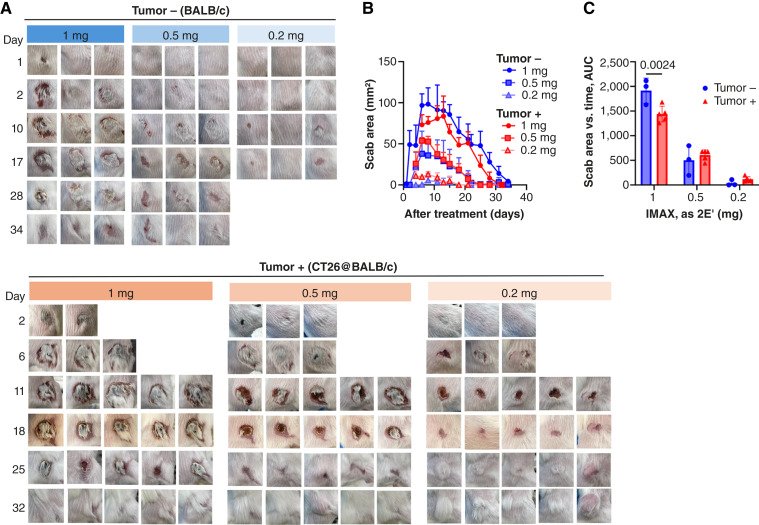
Locally injected IMAX shows dose-dependent skin response in mice. **A,** Scab formation at the injected site after dosing 0.2, 0.5, and 1 mg IMAX (2E′ equivalent) in healthy and CT26 tumor-bearing BALB/c mice (7–8 weeks). *n* = 3 (healthy mice) and 5 (tumor-bearing mice) per group. **B,** Average scab area over time. **C,** Area under the curves (AUC) of scab area vs. time plots in mice receiving different IMAX doses. *P* value was calculated by Sidak multiple comparisons test, following two-way ANOVA. Data are the mean ± SD.

We varied the IMAX dose (equivalent to 2E′ 1, 0.5, or 0.2 mg) to observe its effect on the severity of skin response in healthy BALB/c mice and CT26 tumor-bearing mice (Fig. 4). The skin response was dose-dependent in both groups (Fig. 4A). IMAX (2E′ 1 mg) induced a pronounced and sustained scab formation, with a mean peak scab area of 83.5 ± 24.7 mm^2^ on day 13 in tumor-bearing mice, which resolved in 30 days. Lower doses produced smaller scabs (peak scab area on day 8: 52.8 ± 6.2 mm^2^ for IMAX with 2E′ 0.5 mg and 12.8 ± 3.5 mm^2^ for IMAX with 2E′ 0.2 mg), and they resolved within 20 days. Of note, the overall scab burden (quantified as the area under the curve of scab area vs. time plot) was significantly higher in healthy mice than in tumor-bearing mice (*P* = 0.0024) at the highest dose (Fig. 4B and C), even though local drug retention was lower in healthy skin (Fig. 1). We speculate that the scab formation may have resulted from drug spillage to normal skin tissue, which likely had a more pronounced effect in healthy mice with no tumor to act as a drug reservoir.

We also compared tissue responses to subcutaneously injected 2E′/PTX (binary complex omitting CDN from IMAX) and IMAX in healthy C57BL/6 mice varying the dose (equivalent to 2E′ 1, 0.2, or 0.1 mg). IMAX induced dose-dependent skin responses at the injection site from day 1, aggravating through day 7 (Supplementary Fig. S4), like those in BALB/c mice (Fig. 4). The response to 2E′/PTX seemed to be milder, suggesting that CDN may be the main cause of the skin response.

### IMAX was effective at one-fifth of the initially tested dose in mice

**Figure 5.**
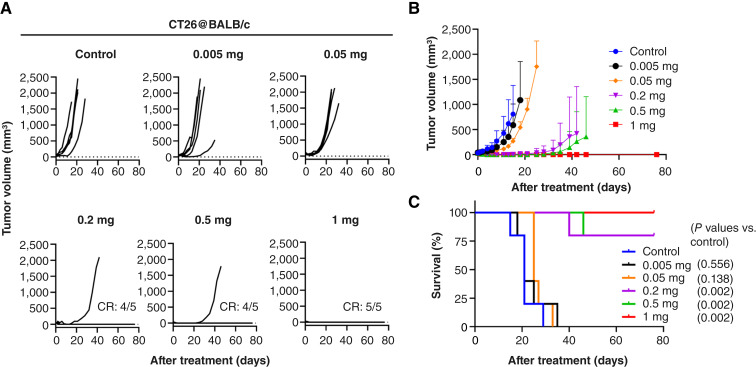
IMAX achieves complete tumor regression at a reduced dose (0.004 mg/mm^3^) in BALB/c mice with CT26 tumor. **A,** CT26 tumors at 30 to 50 mm^3^ were treated with IMAX at concentrations of 1, 0.5, 0.2, 0.05, or 0.005 mg 2E′ equivalent or PBS (control) via intratumoral injection (*n* = 5 per dose). **B,** Average tumor growth curves (mean with SD) and (**C**) survival curves of all groups after treatment. *P* values (numbers in parentheses) were calculated by log-rank (Mantel–Cox) test.

In light of the dose-dependent skin response to IMAX, we examined whether the dose could be reduced without compromising antitumor effect (Fig. 5A and B). In the CT26 tumor model, IMAX containing 2E′ 1 mg, PTX 0.2 mg, and CDN 0.02 mg (typical dose used in earlier studies for various murine tumor models; refs. 2, 8) induced complete tumor regression in all treated mice (five of five), as previously seen (2), for the duration of observation (160 days). Similarly, IMAX led to complete regression in four of five mice at doses equivalent to 2E′ 0.5 and 0.2 mg. Further reductions of IMAX dose (equivalent to 2E′ 0.05 and 0.005 mg) did not delay tumor progression. This result indicates that the IMAX is effective at one-fifth of the originally tested dose in inducing complete tumor regression in 80% of the treated mice (Fig. 5C).

### IMAX was well-tolerated in healthy laboratory dogs

The safety of locally injected IMAX was tested in laboratory beagle dogs. Given that IMAX equivalent to 2E′ 0.2 mg was considered a minimum effective dose for treating 50 mm^3^ in mice (0.004 mg/mm^3^), we used IMAX equivalent to 2E′ 2 mg on one side and IMAX equivalent to 2E′ 1 mg on the other side in each of the first two laboratory dogs (1W and 2P), which would be appropriate for treating 500 and 250 mm^3^ tumors (not considering allometric scaling), respectively. The third laboratory dog (3C) was treated with IMAX equivalent to 2E′ 2 mg on one side.

The IMAX treatments were generally well tolerated with manageable adverse events. VCOG grade 1 to 2 fever was observed after the treatment. The body temperature of the laboratory dogs peaked at 6 to 10 hours after treatment, with a maximum of 103.7°F, and returned to normal temperature 4 hours after the peak. The fever is in line with immunostimulatory functions of IMAX components, such as 2E′ (TLR5 activator) and CDN (STING agonist), which induce production of proinflammatory cytokines and other immune infiltrates (32, 33). VCOG grade 1 to 2 lethargy was observed during the fever, followed by grade 1 lethargy that persisted for 24 to 36 hours after treatment. Neutrophilic leukocytosis was also noted within 24 hours after treatment. The leukocytosis resolved within 7 days after treatment. The administration of dexamethasone (0.1 mg/kg s.c.) and diphenhydramine (2 mg/kg s.c.) after the onset of fever did not seem to alter the course of the fever, as the reaction was similar in dogs receiving the drugs and those not receiving the drugs. Mild discomfort at the injection site was observed and resolved within 1 to 2 days.

### Tumors in companion dogs responded to IMAX treatment

**Figure 6.**
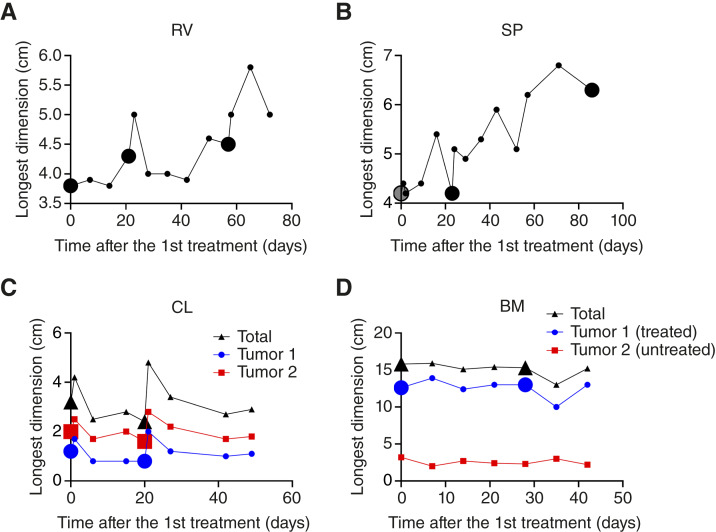
Tumor response to locally injected IMAX in companion dogs. **A,***Dog RV* soft tissue sarcoma. **B,***Dog SP* soft tissue sarcoma. **C,***Dog CL* mammary tumors. **D,***Dog BM* mammary tumors. Larger symbols indicate the times at which IMAX was administered.

As IMAX equivalent to 2E′ 2 mg was well tolerated in all three laboratory dogs, including one with the lowest body weight tested, 7.8 kg, we used IMAX containing 2E′ 2 mg, PTX 0.4 mg, and CDN 0.04 mg per 7.8 kg body weight (corresponding to 2E′ 0.25 mg/kg, PTX 0.05 mg/kg, and CDN 0.005 mg/kg) as the safe dose for the pilot study in companion dogs. We determined the dose based on body weight, expecting that, in the unlikely event that the drug rapidly entered the systemic circulation through tumor vasculature, it would still be tolerable. Table 2 summarizes the information and IMAX regimen for the treated companion dogs. Figure 6 shows tumor size change over time based on caliper measurements, with the best tumor responses summarized in Table 3.

**Table 3.** Tumor response in companion dogs.

Subject - dog	Tumor diagnosis	TNM status	Number of tumors	Best response
RV	STS	T2N0M0	1	iSD
SP	STS	T2N0M0	1	iSD
CL	MCa	T1N0M0	2	iSD
BM	MCa	T3N0M1^a^	2	iSD

Abbreviations: MCa, mammary carcinoma; STS, soft tissue sarcoma; TNM, tumor–node–metastasis.

TNM: T (tumor size: T1 = <3 cm, T2 = 3–5 cm, and T3 = >5 cm); *N* (node status: 0 = none and 1 = present); M (metastasis: 0 = none and 1 = present; TNM classification of tumors in domestic animals. Geneva: World Health Organization; 1980).

a M1 is presumptive, based on imaging only.

Dog RV had one tumor (soft tissue sarcoma, 7.6 cm^3^) and received IMAX by intratumoral injection a total of three times at a dose equivalent to 2E′ 4.4 to 5.6 mg per time. Some leakage occurred at the injection site immediately following the IMAX injection, indicating that the entire dose likely did not stay inside the tumor. The dog’s best overall response was iSD. iCPD was noted at week 9, and the tumor was definitively removed at week 10 via amputation.

Dog SP had one tumor (soft tissue sarcoma, 25.7 cm^3^) and received IMAX intratumoral three times at a dose of 8 to 8.4 mg 2E′. The dog’s best overall response was iSD. iCPD was noted at week 6, and the tumor was definitively removed at week 12 via amputation.

Dog CL had two mammary carcinomas measuring 0.3 and 1 cm^3^, respectively, at the time of the first treatment. The larger tumor (1 cm^3^) was treated with IMAX two times at a dose of 0.6 to 0.9 mg 2E′ equivalent, and the smaller tumor (0.3 cm^3^) was treated two times with IMAX at 0.5 to 0.6 mg 2E′ equivalent. The tumor size increased the day after each treatment but subsided in the following days, suggesting that the temporary increase may have been IMAX-induced swelling as observed in mice. The dog’s best overall response was a 25% reduction in the sum of the longest dimensions of all tumors, which was classified as iSD. At week 7, the tumors were surgically removed via mastectomy while still classified as iSD.

Dog BM had two mammary carcinomas measuring 3.1 cm^3^ (right caudal mammary gland) and 36.3 cm^3^ (left caudal mammary gland), respectively. The larger tumor (36.3 cm^3^) in the left caudal mammary gland was treated with IMAX two times at a dose of 10.8 to 11 mg 2E′ equivalent. The size of both tumors was monitored. The dog’s best overall response was an 18% reduction in the sum of the longest dimensions of the tumors, which was classified as iSD. The tumors were removed at week 6 via mastectomy while still classified as iSD.

These results indicate that IMAX, despite the conservative doses based on body weight, induced measurable responses, especially in dogs with mammary carcinoma. Adverse events included fever (grades 1–2, two dogs), lethargy (grade 1, one dog), pain at injection site (grades 2–3, three dogs), erythema (grade 1, three dogs), swelling (grade 1, three dogs), total bilirubin elevation (grade 3, one dog), bruising (grade 1, two dogs), and dermal nodules/rash (grade 1, two dogs). 

### IMAX induced histologic changes in tumors of companion dogs

**Figure 7.**
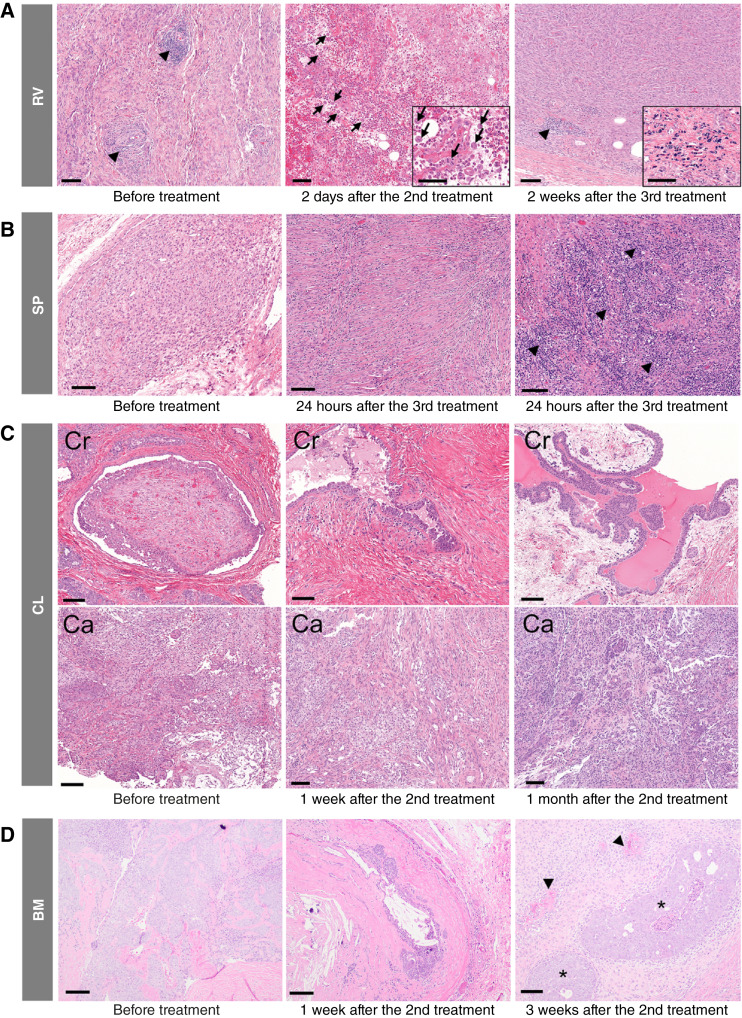
Histology of companion dog tumors before and after IMAX treatment. **A,** Histology of *dog RV* soft tissue sarcoma. Left, perivascular lymphocytic infiltration (▲). Middle, lack of identifiable tumor cells and marked leukocytic (primarily neutrophils) infiltration with mitotically active giant cells (→). Right, perivascular lymphocytic infiltration (▲) and blue extracellular and intracellular granular materials (inset) located mainly at the tumor periphery. **B,** Histology of *dog SP* soft tissue sarcoma. Right, marked intratumoral lymphocytic infiltration (▲). **C,** Histology of *dog CL* mammary carcinomas. Ca, caudal mammary gland; Cr, cranial mammary gland. **D,** Histology of *dog BM* mammary carcinoma. Right, some areas of the tumor 3 weeks after the second treatment include both malignant epithelial (*) component and malignant mesenchymal component with osteoid (▲), characteristic of carcinosarcoma. Scale bars, 100 μm.

The canine tumors were evaluated histologically (Fig. 7) and graded based on established grading schemes for canine soft tissue sarcoma and canine mammary carcinoma, respectively (34, 35). Diagnoses for each biopsy specimen are listed in Table 4.

**Table 4.** Biopsy diagnoses for companion dogs.

Subject - dog	Biopsy 1	Biopsy 2	Biopsy 3
RV	Before treatment Soft tissue sarcoma, grade I	2 days after the second treatment Granulomatous to pyogranulomatous dermatitis with bizarre mitoses	2 weeks after the third treatment Soft tissue sarcoma, grade II^a^
SP	Before treatment Soft tissue sarcoma, grade II	24 hours after the third treatment Soft tissue sarcoma, grade II^a^	Not applicable
CL	Before treatment Cranial: ductal carcinoma, grade I Caudal: complex carcinoma, grade I	1-week after the second treatment Cranial: ductal carcinoma Caudal: complex carcinoma	1-month after the second treatment Cranial: ductal carcinoma, grade I Caudal: complex carcinoma, grade I^a^
BM	Before treatment Solid simple carcinoma, grade III	1-week after the second treatment Carcinoma	3 weeks after the second treatment Grades II and III carcinomas, osteosarcoma features, carcinosarcoma^a^

a Denotes final biopsies obtained from definitive surgeries to remove the tumors (RV, SP: amputated limbs; CL, BM: mastectomies).

Dog RV was initially diagnosed with a grade I (low grade) soft tissue sarcoma. In the pretreatment specimen, small to moderate numbers of lymphocytes, plasma cells, and eosinophils were observed around multiple intratumoral and peritumoral blood vessels (Fig. 7A). Neoplastic cells did not immunolabel with 2′,3′-cyclic nucleotide 3′-phosphodiesterase (CNPase), S100, or smooth muscle actin IHC antibodies, ruling out peripheral nerve sheath tumor and leiomyosarcoma. In the biopsy obtained 2 days after the second treatment, extensive necrosis and hemorrhage were present, and neoplastic cells were not definitively identified in the examined sections. A few small clusters of elongate to spindled cells were admixed with inflammatory cells. Within the dermis, a marked neutrophilic and histiocytic infiltration was observed, mixed with fibrin and necrotic cell debris. Throughout the section were numerous large round cells exhibiting bizarre mitoses [Fig. 7A (middle), indicated by arrow]. IHC for CD3 revealed a low number of T cells with strong membranous staining within the superficial and deep dermal connective tissues, among numerous macrophages with moderate to strong cytoplasmic ionized calcium-binding adapter molecule 1 (Iba-1) staining and strong cytoplasmic vimentin staining. The large cells interpreted as bizarre mitotic figures on H&E were negative for CD3, Iba-1, and cytokeratins but positive for vimentin (Supplementary Fig. S5), suggesting mesenchymal origin. The third biopsy was obtained at the time of limb amputation, 2 weeks after the third treatment. The tumor had recurred as a large, highly cellular neoplasm diagnosed as a grade II (medium grade) soft tissue sarcoma (Fig. 7A). At the margin of one section was an area of locally extensive granulomatous to pyogranulomatous inflammation, including a few multinucleated giant cells containing nonstaining intracytoplasmic crystalline material interpreted as suture material. Adjacent to this area, and deeper in the mass, was an area of extensive necrosis and hemorrhage, surrounded by aggregates of lymphocytes and fewer plasma cells, and a focus of large macrophages occasionally containing nonstaining crystalline material (suture). Within areas of necrosis, numerous macrophages contained intracytoplasmic, deep blue, granular material (Fig. 7A).

Dog SP was initially diagnosed with a grade II soft tissue sarcoma. In the pretreatment biopsy, the inflammatory component was minimal, consisting mainly of scattered intratumoral and peritumoral CD18^+^ macrophages (Fig. 7B). The second biopsy (24 hours after the third treatment) was obtained at the time of limb amputation, and the diagnosis was again grade II soft tissue sarcoma. Being a surgical biopsy, this biopsy was much larger than the pretreatment biopsy and better indicated the aggressive nature of the tumor. There was an area of suspected tumor loss consisting of extensive necrosis and granulocytic infiltration (Fig. 7B). Moderate to marked numbers of lymphocytes infiltrated the neoplasm. Numerous small lymphocytes and fewer plasma cells, which occasionally formed small aggregates often in perivascular areas, were suggestive of tumor regression (Fig. 7B). Multifocally within the mass were areas of necrosis with infiltration by neutrophils and fewer macrophages, erythrocytes, and hemosiderophages mixed with cell debris (Fig. 7B).

Dog CL was initially diagnosed with a grade I (low grade) mammary ductal carcinoma in the cranial gland and a grade I mammary complex carcinoma in the caudal gland. In the second specimen (1-week after the second treatment), a few ill-defined neoplastic epithelial nests within a dense fibrous stromal and mild peritumoral lymphoplasmacytic infiltration remained, indicative of a potentially regressing tumor (Fig. 7C). Neoplastic cells had a mostly sarcomatoid morphology with a focal squamous nodule (Fig. 7C). Histologic findings were similar in the third biopsy specimen obtained at 1 month after the second treatment, with mostly sarcomatoid and rare squamous nests of neoplastic cells (Fig. 7C). In the cranial mass, lymphocytes, fewer plasma cells, and rare mast cells surrounded some of the neoplastic ducts (Fig. 7C). Overall, the posttreatment biopsies showed morphologic alterations in tumor cells, reduced tumor density, and the presence of inflammatory infiltrates.

Dog BM was initially diagnosed with a grade III (high grade) solid carcinoma in the left caudal mammary gland (Fig. 7D). Moderate mitotic activity and a very mild, mixed cellular peritumoral inflammatory reaction were noted. A second mass was present in the right caudal mammary gland and was confirmed to be a grade II ductal mammary carcinoma at the time of mastectomy. This second mass was not treated with IMAX but monitored. The second biopsy of the treated mass in the left caudal mammary gland was obtained 1 week after the second treatment (Fig. 7D). The tumor had become a multinodular, collagen-rich, and multifocally mineralized mass, with only small regions of neoplastic cells, consistent with tumor regression. Lymphoplasmacytic inflammation with formation of lymphoid follicles was present in the surrounding tissues. The third biopsy (3 weeks after the second treatment) was obtained at the time of mastectomy. Different regions of the left caudal mammary mass were classified as ductal mammary carcinoma grade II and simple mammary carcinoma grade III. The center of the left caudal mass had become mineralized and acquired features of a malignant mesenchymal neoplasm similar to those found in osteosarcoma. The mixed malignant epithelial and mesenchymal neoplastic characteristics were indicative of a carcinosarcoma (Fig. 7D). An inflammatory reaction was mostly absent.

Overall, these histologic changes in the canine tumors were consistent with those in mice, represented by infiltration of innate immune cells, including neutrophils, macrophages, and lymphocytes, as well as tumor necrosis.

### RNA-seq analysis of companion dog tumor biopsies suggested early upregulation of the innate immune system by IMAX

**Figure 8.**
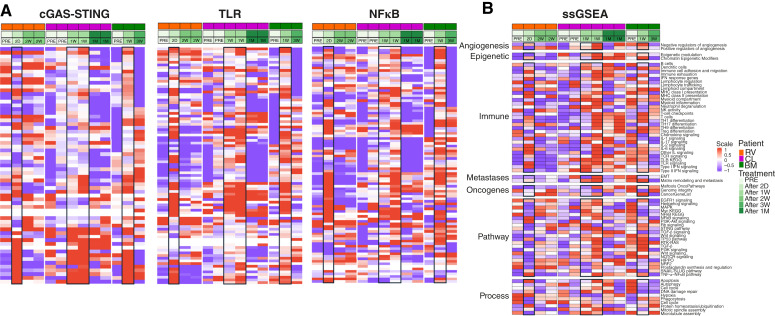
RNA-seq of companion dog tumor biopsies before and after IMAX treatment. **A,** Heatmaps of differentially expressed genes in cGAS/STING pathway, TLR pathway, and NFkB pathway. Data were scaled from positive to negative gene expression values to evaluate trends before and after IMAX treatment. **B,** A z-scaled heatmap of the ssGSEA scores for gene sets in specific biological pathways. The color scale indicates z-scaled ssGSEA scores across samples. *Dog RV*: PRE (before treatment), 2D (2 days after the second treatment), and 2W (2 weeks after the third treatment, two locations from the same mass). *Dog CL*: PRE (before treatment, two locations, cranial and caudal masses), 1W (1 week after the second treatment, two locations), and 1M (1 month after the second treatment, two locations). *Dog BM*: PRE (before treatment), 1W (1 week after the second treatment), and 3W (3 weeks after the second treatment). EMT, epithelial–mesenchymal transition; KEGG, Kyoto Encyclopedia of Genes and Genomes; TCR, T-cell receptor; Treg, regulatory T cell.

To complement the histologic evaluation, we performed RNA-seq analysis on biopsies from the dogs RV (one location before treatment and two locations after treatment within the same mass), CL (two locations, cranial and caudal tumors), and BM (one location). Figure 8A summarizes the changes of treated tumors in gene expression relevant to cGAS-STING, TLR, and NFκB pathways before and after IMAX treatment. All three dogs showed early upregulation of most genes within these pathways following IMAX treatment (2 days for RV and 1 week for CL and BM) compared with before treatment, consistent with histologic evidence of innate immune activation. At later time points (2 weeks for RV, 1 month for CL, and 3 weeks for BM) after IMAX treatment, gene expression returned to baseline, showing patterns similar to before treatment. ssGSEA analysis (Fig. 8B) shows notable upregulation of gene set profiles associated with increased immune cell infiltration (dendritic cells, T cells, B cells, Th cells, NK cells, and neutrophils) and tumor immunogenicity (MHC I/II presentation, IL/IFN signaling, TLR signaling, NFκB signaling, and STING pathway) in the 1 week post-IMAX treatment biopsies of dog CL, who had reduction in tumor size. These trends suggest that local IMAX treatment induced early upregulation of genes relevant to innate immune activation.

## Discussion

Our previous studies in multiple mouse tumor models have demonstrated that locally injected IMAX prevented tumor growth, resulting in tumor-free survival in most treated mice (2, 8). In this study, we investigated the trajectory of local events at both gross and cellular levels, assessed the safety of local IMAX treatment in mice and dogs, and evaluated its feasibility for controlling tumors in larger animals, i.e., dogs. Our results show that IMAX is retained at the injection site, where it triggers a rapid influx of innate immune cells, which translates to antitumor effects in mice and dogs with manageable side effects.

IMAX consists of 2E′, a derivative of polyethyleneimine, which encapsulates PTX in the hydrophobic core and carries CDN on the surface via electrostatic interaction. Due to the polyethyleneimine component, IMAX assumes a positive surface charge (+19 mV; ref. 8), which contributes to favorable interactions with cells in the tumor microenvironment (2, 8). However, the positive charge interferes with its systemic delivery to tumors due to uncontrolled interactions with serum proteins, resulting in aggregation, instability, and rapid clearance by the mononuclear phagocytic system (36–38). Therefore, we have administered IMAX by local injection with the intent to treat accessible tumors. Intratumoral injection is a clinically relevant and feasible approach, supported by the FDA-approved intralesional cancer therapy (39) and ongoing clinical trials of local immunotherapy (40).

One of the expected benefits of local delivery of immunotherapy is the potential to avoid systemic absorption of cytotoxic drugs (PTX) and immunostimulants (CDN) and maximize their local activities. Several local drug delivery systems have been reported in the literature for a similar purpose, such as injectable hydrogel (41, 42), polymeric nanoparticles (43, 44), and polymeric implants (45). These systems prolonged local retention and sustained drug release, resulting in improved regional immune activation and antitumor efficacy after intratumoral administration. Consistently, we observe that locally injected IMAX was retained in the tumor for at least 48 hours in mice, with minimal systemic absorption (Fig. 1). Local retention of active components led to rapid changes in the cell population. Histologic evaluation of biopsies obtained before and after treatment indicated infiltration of neutrophils, macrophages, and lymphocytes following IMAX injection with locally extensive tumor necrosis, indicative of tumor regression. The cellular events were also reflected in gross skin responses, including locally extensive erythema and crusting (scabbing) with edema and necrosis of the surrounding subcutaneous connective tissue, skeletal muscle, and skin. Tumors regressed visibly within 1 day and were replaced by scabs within 1 to 2 weeks in mice.

The infiltration of innate immune cells indicates that IMAX exerts immunostimulatory effects, consistent with the known roles of its components. The comparison between IMAX and a binary complex omitting CDN (Supplementary Fig. S4) suggests that CDN may be the primary driver of the observed skin response. As a STING agonist, CDN stimulates multiple cells in the tumor microenvironment, including APCs and endothelial cells (7, 46). In particular, STING activation in tumor endothelial cells increases vascular permeability and the expression of endothelial adhesion molecules, such as E-selectin, intercellular adhesion molecule 1, and vascular cell adhesion molecule 1, enhancing immune cell infiltration into the tumor site (47). The edema, erythema, and immune cell infiltration following local injection of IMAX are consistent with these functions of CDN in endothelial cells.

The scab-covered skin was restored to a normal outlook with fur in 30 days; thereafter, most mice remained tumor-free over the duration of observation (160 days). Therefore, we consider the scab formation a sign of a positive tumor response to the treatment. Nevertheless, it would be desirable to minimize tissue irritation, which may lead to open wounds prone to infection. To this end, we could reduce the dose to one-fifth of the initial dose (equivalent to 2E′ 0.2 mg per mouse for treating 50 mm^3^ mouse tumor), while maintaining the antitumor effect with minimal tissue responses in mice (Fig. 5).

The effects of the IMAX treatment in the companion dogs as related to change in tumor size over time and histologic features were especially encouraging for two reasons. First, the doses of IMAX used were relatively low. The IMAX doses in the companion dogs were selected based on body weight, with safety as a high priority. Although systemic drug absorption was not observed in the mouse studies (Fig. 1), it was still considered possible for the tested dogs, because naturally occurring tumors in dogs and humans can have extensive and leaky vasculatures. The laboratory dogs (7.8–11.4 kg) received IMAX at a dose equivalent to 2E′ ≤ 2 mg per administration, which was well-tolerated with manageable adverse events. Not knowing whether the effective doses in mice would be required in the canine tumors, we kept the IMAX dose for companion dogs at the highest dose confirmed to be safe in the laboratory dogs. For the companion dogs treated based on body weight, the IMAX doses ranged from 1.2 mg (0.6 mg per tumor × two tumors in a dog) to 11 mg of 2E′ per administration in the dogs weighing 5 to 43.2 kg. According to the results in mice, however, these doses would correspond—without allometric scaling—to the doses used to treat tumor volumes of 0.15 to 2.8 cm^3^, which were substantially smaller than the tumors in the companion dogs (0.3–36.3 cm^3^). Second, naturally occurring canine cancers often show extensive heterogeneity and innate and acquired resistance to immunotherapies and other drugs; thus, canine cancers are much more difficult to control than the experimentally induced, more homogeneous tumors in rodents. The less favorable response to intratumoral immunotherapies in dogs compared with responses in mice has been reported in multiple studies evaluating a slow-release IL-12 formulation (48), an IL-2/agonist anti-CD40 antibody (49), viral-related products (50, 51), and an intratumoral mycobacterial approach (52). Despite the relatively low doses and complex cancers treated, IMAX showed measurable antitumor activity in mammary carcinomas, in that each carcinoma measured smaller after treatment, although tumor response was classified as iSD without meeting the criteria for iPR. Histologically, IMAX effects in the companion dogs were notable and similar to the effects in mice, with local immune cell infiltration, necrosis, and an initial reduction in tumor size. RNA-seq analysis of tumor biopsies showed early upregulation of innate immune activities following IMAX treatment, consistent with histologic evidence of innate immune cell infiltration.

### Conclusion

This study demonstrates the feasibility and safety of locally delivered IMAX, a nanoparticulate complex of immunoactive polymer (2E′), PTX, and CDN, for treating experimental tumors in mice and spontaneously occurring tumors in dogs as steps toward translation to human application. Consistent with previously reported antitumor activity, IMAX remained localized at the injection site and induced rapid infiltration of innate immune cells into murine tumors. Laboratory dogs tolerated IMAX with manageable adverse events. Despite conservative dosing, IMAX elicited measurable antitumor effects in companion dogs with mammary carcinoma. Although tumor shrinkage did not meet partial response criteria, histologic and transcriptomic evidence of innate immune activation shown in dogs supports the therapeutic potential of IMAX as a local chemoimmunotherapy. Future studies should focus on optimizing doses and treatment schedules and on integrating with other therapeutic modalities to further advance clinical translation.

## Supplementary Material

Supplementary Fig. 1 Locally injected IMAX is retained at the injection site with minimal systemic absorption

Supplementary Fig. 2 Skin histology of healthy or tumor-bearing mice pre- and post-IMAX treatment (low mag)

Supplementary Fig. 3 Gram-stained section of blue granular material in the injection site (tumor)

Supplementary Fig. 4 Locally injected 2E'/PTX and IMAX show dose-dependent skin responses in mice

Supplementary Fig. 5 Immunohistochemistry (IHC) of RV soft tissue sarcoma 2 days post IMAX treatment

Supplementary Table 1 Antibodies used in IHC of canine tissues

## Data Availability

The data that support the findings of this study as well as the materials (2E′ and IMAX) are available from the corresponding authors upon reasonable request. RNA-seq data generated in this study is available at the NCBI Gene Expression Omnibus under accession GSE317158. All other raw data generated in this study are available upon request from the corresponding authors.
