# Supplementary material for: Nanoparticulate Immunoactive Complex for Local Chemoimmunotherapy: From Murine Models to Pilot Canine Study
Source: Cancer Res Commun. 2026 Jun 22;6(6):1455–69. doi: 10.1158/2767-9764.CRC-26-0110 (PMC13285167; doi:10.1158/2767-9764.CRC-26-0110)
Supplement: Supplementary Fig. 1 — Locally injected IMAX is retained at the injection site with minimal systemic absorption [file crc-26-0110_supplementary_fig.1_suppsf1.pdf]

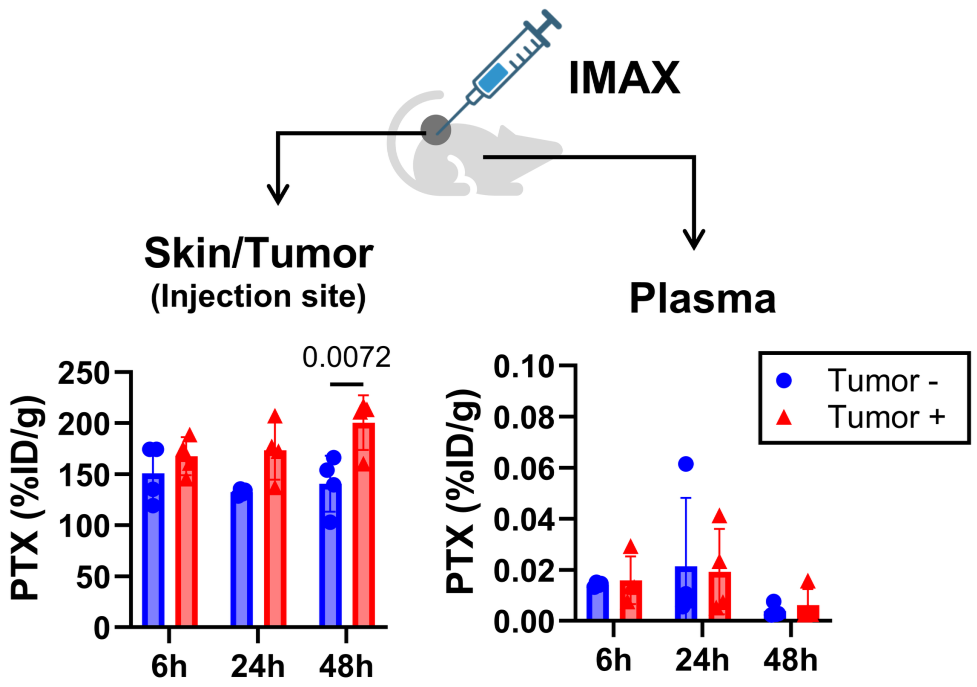

Supplementary Fig. 1. Locally injected IMAX is retained at the injection site with minimal systemic absorption. PTX levels (%injected dose/g) in skin (or tumor) and plasma at 6, 24, and 48 hours after SC or IT injection of IMAX (containing 1 mg 2E') in healthy and CT26 tumor-bearing BALB/c mice (7-8 weeks). n=4 mice per time point. Data are mean ± SD. P-value was calculated by Sidak's multiple comparisons test, following two-way ANOVA.
