# Supplementary material for: Nanoparticulate Immunoactive Complex for Local Chemoimmunotherapy: From Murine Models to Pilot Canine Study
Source: Cancer Res Commun. 2026 Jun 22;6(6):1455–69. doi: 10.1158/2767-9764.CRC-26-0110 (PMC13285167; doi:10.1158/2767-9764.CRC-26-0110)
Supplement: Supplementary Fig. 2 — Skin histology of healthy or tumor-bearing mice pre- and post-IMAX treatment (low mag) [file crc-26-0110_supplementary_fig.2_suppsf2.pdf]

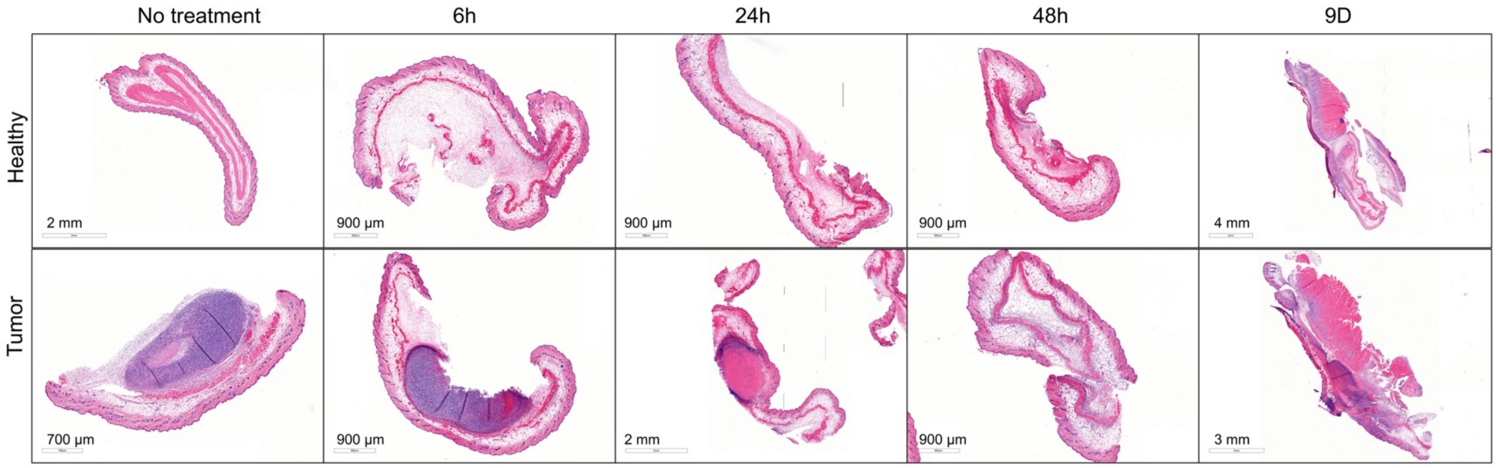

Supplementary Fig. 2. Skin histology of healthy or tumor-bearing mice pre- and post-IMAX treatment shown at low magnifications.
