# Supplementary material for: Nanoparticulate Immunoactive Complex for Local Chemoimmunotherapy: From Murine Models to Pilot Canine Study
Source: Cancer Res Commun. 2026 Jun 22;6(6):1455–69. doi: 10.1158/2767-9764.CRC-26-0110 (PMC13285167; doi:10.1158/2767-9764.CRC-26-0110)
Supplement: Supplementary Fig. 3 — Gram-stained section of blue granular material in the injection site (tumor) [file crc-26-0110_supplementary_fig.3_suppsf3.pdf]

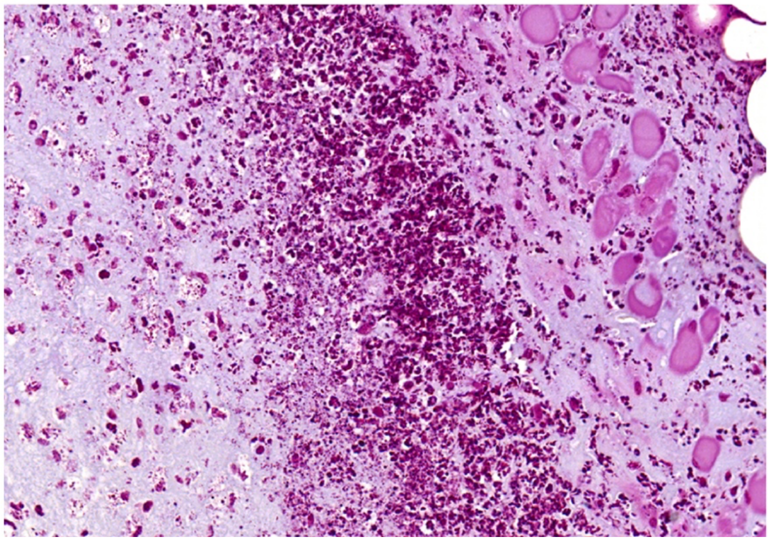

Supplementary Fig. 3. Representative Gram-stained section of blue granular material in the injection site (tumor) of a CT26 tumor-bearing BALB/c mouse at 9 days post-IMAX treatment.
