# Supplementary material for: Nanoparticulate Immunoactive Complex for Local Chemoimmunotherapy: From Murine Models to Pilot Canine Study
Source: Cancer Res Commun. 2026 Jun 22;6(6):1455–69. doi: 10.1158/2767-9764.CRC-26-0110 (PMC13285167; doi:10.1158/2767-9764.CRC-26-0110)
Supplement: Supplementary Fig. 4 — Locally injected 2E'/PTX and IMAX show dose-dependent skin responses in mice [file crc-26-0110_supplementary_fig.4_suppsf4.pdf]

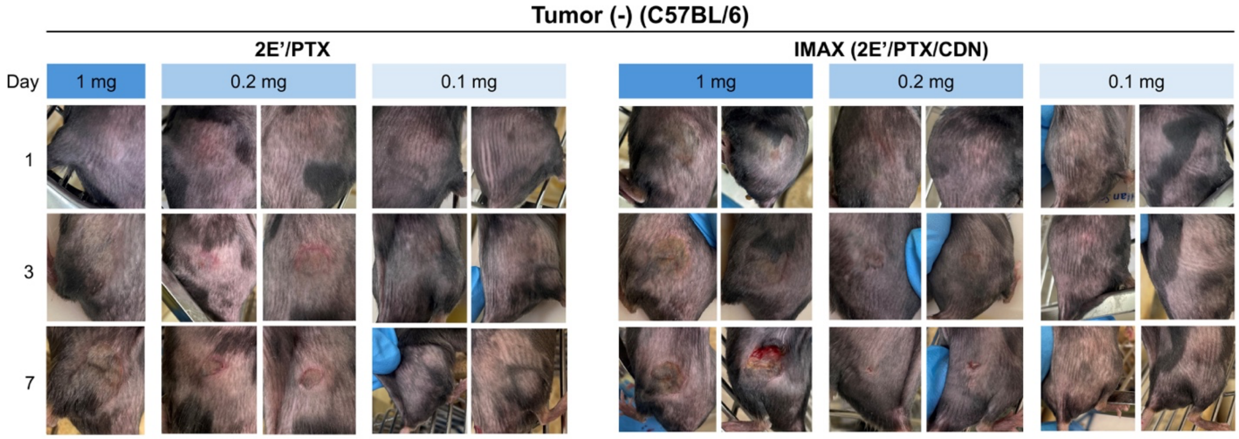

Supplementary Fig. 4. Locally injected 2E'/PTX and IMAX show dose-dependent skin responses in mice. Healthy C57BL/6 male mice (40 weeks old) were administered with 2E'/PTX (5:1 w/w) or IMAX (5:1:0.1 w/w/w) via SC injection at concentrations of 1, 0.2, or 0.1 mg 2E' equivalent (n=1-2 per group). Scab formation at the injection site was monitored on 1, 3, and 7 days after injection.
