# Supplementary material for: Nanoparticulate Immunoactive Complex for Local Chemoimmunotherapy: From Murine Models to Pilot Canine Study
Source: Cancer Res Commun. 2026 Jun 22;6(6):1455–69. doi: 10.1158/2767-9764.CRC-26-0110 (PMC13285167; doi:10.1158/2767-9764.CRC-26-0110)
Supplement: Supplementary Fig. 5 — Immunohistochemistry (IHC) of RV soft tissue sarcoma 2 days post IMAX treatment [file crc-26-0110_supplementary_fig.5_suppsf5.pdf]

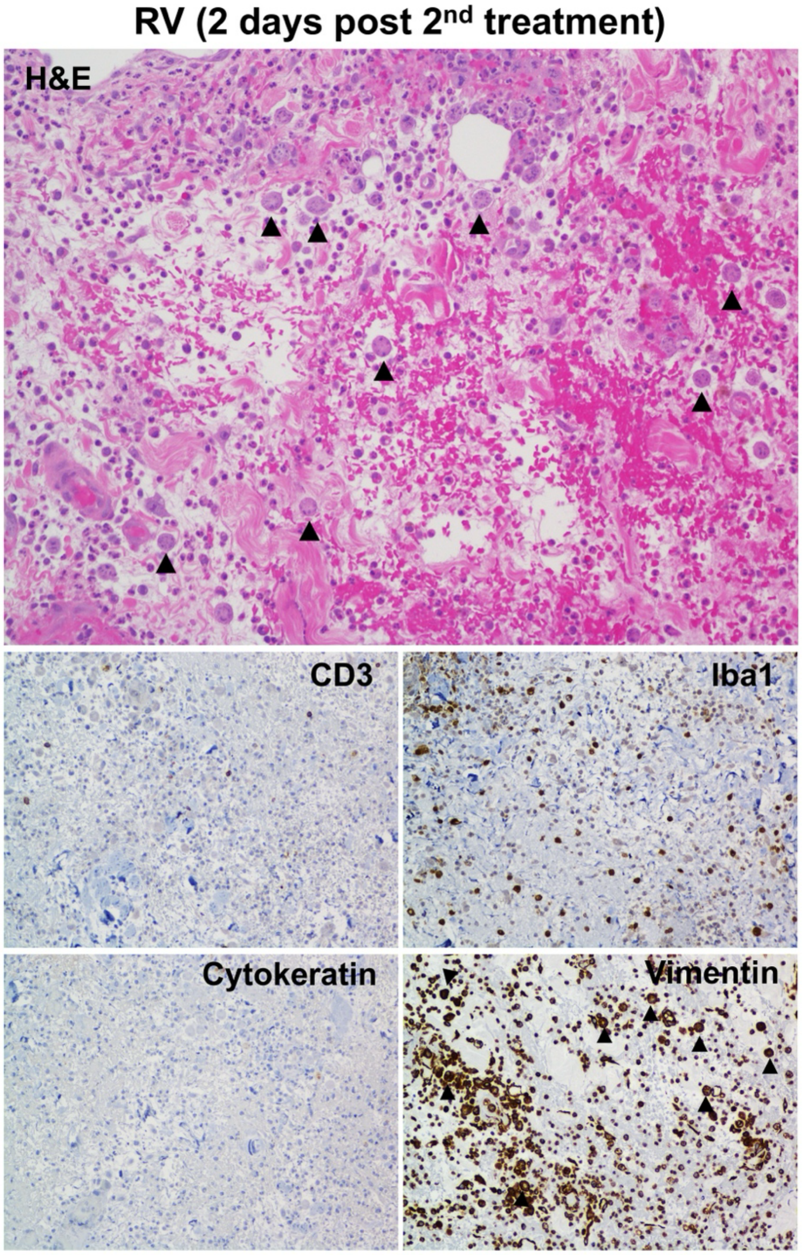

Supplementary Fig. 5. Immunohistochemistry (IHC) of RV soft tissue sarcoma 2 days post IMAX treatment. H&E, CD3, Iba1, Cytokeratin, and Vimentin IHC 2 days post first IMAX treatment. Note large, bizarre cells (▲) labeling with vimentin (lower right).
