# Supplementary material for: Nanoparticulate Immunoactive Complex for Local Chemoimmunotherapy: From Murine Models to Pilot Canine Study
Source: Cancer Res Commun. 2026 Jun 22;6(6):1455–69. doi: 10.1158/2767-9764.CRC-26-0110 (PMC13285167; doi:10.1158/2767-9764.CRC-26-0110)
Supplement: Supplementary Table 1 — Antibodies used in IHC of canine tissues [file crc-26-0110_supplementary_table_1_suppst1.pdf]

Supplementary Table 1. Antibodies used in IHC of canine tissues

Antibody	Vendor	Clone	Dilution	Pre-treatment	Host	Incubation	Chromagen
CD3	Dako	NA	1:100	DIVA HIER	Rabbit	30 min RT	DAB
CD11d	Dr. Moore-UCD	CA18.3C6	1:200	DIVA HIER	Mouse	60 min RT	DAB
CNPase	BioLegend	SMI-91	1:2000	DIVA HIER	Mouse	60 min RT	DAB
S100	Dako	Ig0EO	Ready to use	Reveal HIER	Rabbit	30 min RT	DAB
SMA	Dako	1A4	1:200	No treatment	Mouse	45 min RT	DAB
Vimentin	Zeta Corp	ZR381	Ready to use	DIVA HIER	Rabbit	30 min RT	DAB
Cytokeratins	Dako	AE1/AE3	1:100	DIVA HIER	Mouse	60 min RT	DAB
Iba-1	Biocare	NA	1:400	DIV HIER + pepsin	Rabbit	60 min RT	DAB

DIVA: Diva Decloaker (Biocare Medical, Pacheco, CA); HEIR: heat-induced epitope retrieval; RT: room temperature; DAB: diaminobenzidine; CNPase: 2′,3′-cyclic nucleotide 3′-phosphodiesterase; SMA: Smooth muscle actin; Iba-1: Ionized calcium-binding adaptor molecule 1
